# Corticosteroids in Pediatric Septic Shock: A Narrative Review

**DOI:** 10.3390/jpm14121155

**Published:** 2024-12-17

**Authors:** Immacolata Rulli, Angelo Mattia Carcione, Federica D’Amico, Giuseppa Quartarone, Roberto Chimenz, Eloisa Gitto

**Affiliations:** 1 Neonatal and Pediatric Intensive Care Unit, University Hospital of Messina, 98124 Messina, Italy; angelo.carcione@studenti.unime.it (A.M.C.); federica.damico1@studenti.unime.it (F.D.); giuseppa.quartarone@polime.it (G.Q.); egitto@unime.it (E.G.); 2 Pediatric Nephrology with Dialysis Unit, Maternal-Infantile Department, University Hospital of Messina, 98124 Messina, Italy; rchimenz@unime.it

**Keywords:** septic shock, steroids, children, mortality, severity

## Abstract

**Objective:** A controversial aspect of pediatric septic shock management is corticosteroid therapy. Current guidelines do not recommend its use in forms responsive to fluids and inotropes but leave the decision to physicians in forms refractory to the first steps of therapy. **Data Sources:** Review of literature from January 2013 to December 2023 from online libraries Pubmed, Medline, Cochrane Library, and Scopus. **Study Selection:** The keywords “septic shock”, “steroids” and “children” were used. **Data Extraction:** Of 399 articles, 63 were selected. **Data Synthesis:** Regarding mortality, although the 2019 Cochrane review supports reduced mortality, benefits on long-term mortality and in patients with CIRCI (critical illness-related corticosteroid insufficiency) are not clear. Yang’s metanalysis and retrospective studies of Nichols and Atkinson show no difference or even an increase in mortality. Regarding severity, the Cochrane review claims that hydrocortisone seems to reduce the length of intensive care hospitalization but influences the duration of ventilatory and inotropic support, and the degree of multi-organ failure appears limited. Further controversies exist on adrenal function evaluation: according to literature, including the Surviving Sepsis Campaign guidelines, basal or stimulated hormonal dosages do not allow the identification of patients who could benefit from hydrocortisone therapy (poor reproducibility). Regarding side effects, muscle weakness, hypernatremia, and hyperglycemia are the most observed. **Conclusions:** The literature does not give certainties about the efficacy of corticosteroids in pediatric septic shock, as their influence on primary outcomes (mortality and severity) is controversial. A subgroup of patients suffering from secondary adrenal insufficiency could benefit from it, but it remains to be defined how to identify and what protocol to use to treat them.

## 1. Introduction

Septic shock is a major cause of both mortality and morbidity in children worldwide, particularly in low- and lower middle-income countries (LICs and LMICs), which account for 80% of cases and deaths globally [1,2]. Early treatment generally involves fluid resuscitation to ensure adequate organ perfusion [3]. Additionally, hemodynamic support with vasoactive agents is a critical component in managing patients with fluid-refractory septic shock (FRSS). For these patients, the Surviving Sepsis Campaign guidelines [4] recommend considering corticosteroids as an adjunctive therapy. However, the use of adjunctive corticosteroids varies significantly in clinical practice, with recommendations often described as “may or may not be used”.

Gene expression analyses in children with sepsis have identified endotypes associated with either increased or decreased mortality risk with corticosteroid treatment. Despite these findings, the true effect of corticosteroid therapy on sepsis-related mortality and morbidity remains uncertain due to the lack of large, well-designed pediatric interventional trials, although some are currently underway (e.g., NCT0340139).

Corticosteroids may improve hemodynamics in patients with septic shock through various mechanisms, including enhancing myocardial contractility and vasomotor tone by increasing calcium availability, increasing β-adrenergic receptor sensitivity and expression, reducing norepinephrine reuptake, and decreasing the production of nitric oxide and prostacyclin. Additionally, corticosteroids may stimulate intracellular adhesion factors, which could play a role in reducing capillary leak.

In the literature, two opposing perspectives on corticosteroid use in pediatric septic shock have emerged, supported by both clinical and pathophysiological arguments. Proponents of hydrocortisone highlight its pleiotropic effects, which contribute to hemodynamic stabilization through both immediate nongenomic mechanisms—such as decreased norepinephrine reuptake, increased β-adrenergic receptor sensitivity, and enhanced calcium availability in myocardial and vascular smooth muscle cells, resulting in inotropic and vasoconstrictive effects—and delayed genomic mechanisms, including reduced production of prostacyclins and nitric oxide, increased vascular tone, and stimulation of intercellular adhesion factors, which reduce capillary leak and enhance inotropic effects [5].

Conversely, critics of hydrocortisone in septic shock raise concerns about its immunosuppressive effects, particularly on an already compromised adaptive immune system in pediatric sepsis. Cortisol can impair T-cell receptor signalling, T and B lymphocyte function, and antigen presentation. Furthermore, potential corticosteroid side effects, such as hyperglycemia, delayed wound healing, muscle weakness, and increased risk of infections, are particularly significant in critically ill children. Exogenous steroids may also exacerbate the sepsis-induced stress response, increasing the risk of metabolic derangements and progression to multi-organ failure (MOF) [6].

Given these conflicting perspectives, we conducted a systematic review and meta-analysis in children with FRSS to evaluate the effects of specific vasoactive agents on all-cause mortality and other clinically important outcomes.

## 2. Materials and Methods

The research question has been resumed in a PICO (population, intervention, comparison, outcomes) format. The population is represented by children affected by septic shock and subjected to steroid therapy to evaluate the outcome of mortality, morbidity, and side effects compared to those who have not practiced this kind of therapy.

### 2.1. Inclusion Criteria

(a) Studies on patients (age less or equal to 18 years) affected by refractory septic shock who received steroid treatment.
(b) Literature published from January 2013 to December 2023.
(c) To clear up such controversial questions, we decided to select studies from different levels of evidence (RCT and observational cohort studies included).

### 2.2. Exclusion Criteria

a. studies not pertaining to septic shock
b. studies just on adult patients
c. studies on preterm newborns
d. not in the English language
e. abstracts
f. book chapters
g. conference abstracts
h. preprint articles

### 2.3. Search Strategy

**Figure 1:**
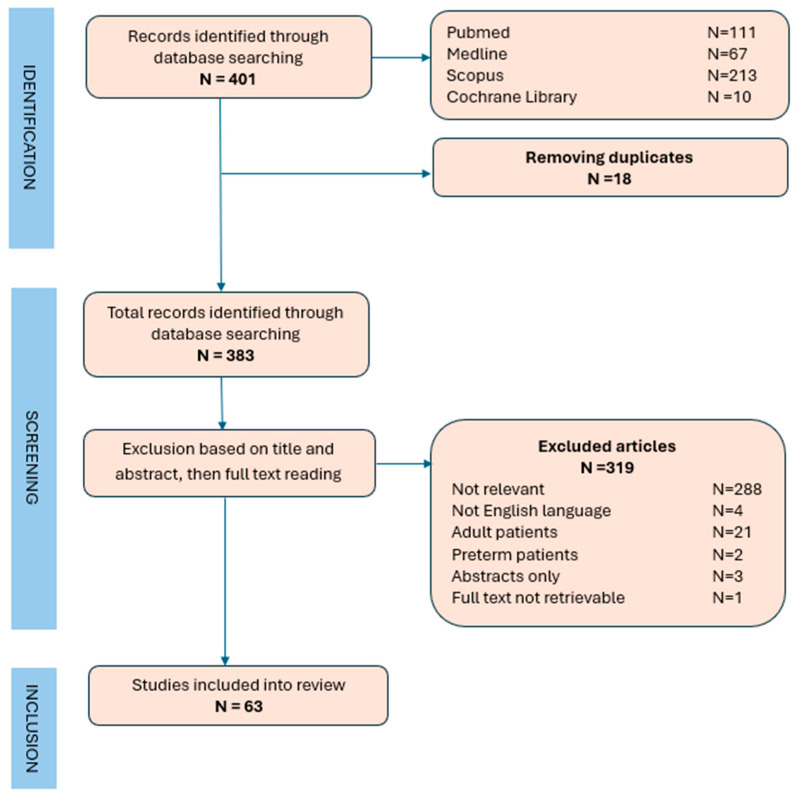
Flowchart for included and excluded studies.

A narrative review of the literature was conducted, searching the online libraries Pubmed, Cochrane Library, Medline, and Scopus. The keywords “septic shock”, “steroids”, and “children” were used for the research. Figure 1.

### 2.4. Outcomes

Our primary outcome was to determine if steroid therapy in refractory pediatric septic shock was associated with a reduction or an increase in mortality.

Secondary outcomes were shock reversal time, severity and duration of MOF, duration of inotropic support, duration of ventilatory support, PICU, and hospital length of stay.

### 2.5. Selected Articles

We identified 401 eligible studies through the online database search strategy. After removing duplicates, we read titles and abstracts to assess eligibility for the review; the potentially relevant ones were read in full text. At the end of this screening, 63 studies were selected.

## 3. Results

### 3.1. Mortality

The 2019 Cochrane review, including studies on both adults and children, states that subgroup analysis based on participant-related factors suggests that age (children versus adults) does not affect the patient’s response to corticosteroids (hydrocortisone or equivalent dose of another steroid). Corticosteroids probably reduce 28-day mortality compared to placebo or standard therapy (relative risk RR 0.91, with 95% confidence interval between 0.84 and 0.99, 11.233 participants, 50 studies, evidence of moderate certainty), with little/no difference in long-term mortality (RR 0.97, CI 95% from 0.91 to 1.03, 6236 participants, seven studies, evidence of poor certainty). They also seem to reduce hospital mortality (RR 0.90, CI 95% 0.82–0.99, 8183 participants, 26 studies, moderate degree of certainty). Children with septic shock may also gain more survival benefits from steroid therapy than those with less severe sepsis. Patients with critical adrenal cortical insufficiency (CIRCI) seem to have no significant reduction in the 28-day risk of death [7].

Yang et al. conducted a meta-analysis including 12 RCT studies (701 children recruited, suffering from shock caused by sepsis or dengue fever). The included studies were divided into a first group (1975–1996) and a second (2009–2017). Older studies were conducted in Asia and Africa, most recent instead in high resource countries like the UK and Canada. Steroids considered were hydrocortisone, methylprednisolone, and dexamethasone. The start dose of hydrocortisone was between 2 and 37.5 mg/kg. The length of therapy was between 1 and 7 days. Regarding the primary outcome, the fixed effect model showed reduced mortality in patients treated compared to the controls (odds ratio: 0.67; 95% confidence interval: 0.46–0.98; *p* = 0.041), but applying a random effect model results exhibited a negative result (odds ratio: 0.69; 95% confidence interval: 0.32–1.51; *p* = 0.252). Dose-response analysis showed also that a low dosage (0.6–5 mg/kg) could reduce mortality, a high dosage (5–37.5 mg/kg) could increase it instead. The authors suggested against corticosteroid application for septic shock in children under current medical conditions. However, several limitations affected this study: the study level, the low number of septic shock patients, the analysis of just about mg/kg dose and not about total dose, and the limited data about the follow-up period [8].

Nichols et al. conducted a retrospective chart review recruiting 70 children with refractory septic shock. Those were subjected to a dosage of total random serum cortisol and treated with stress-dose hydrocortisone in a retrospective study. Children treated had a higher mortality in PICU and a more severe degree of disease control (all *p* < 0.05). In children with serum cortisol > 18 mcg/dl, corticosteroid therapy was associated with increased mortality [9].

Atkinson et al. conducted a retrospective analysis of an ongoing multicenter database. Using PERSEVERE, a validated stratification tool based on biomarkers, 496 patients were stratified into three groups of initial mortality risk (low, intermediate, and high). Among corticosteroids, 78% received hydrocortisone, 16% methylprednisolone, and 6% dexamethasone. The median duration of corticosteroids was 5 days. Patients receiving corticosteroids in the first 7 days after hospitalization (n = 252) were compared with those who did not receive them (n = 244). Children treated had a higher mortality rate than those not treated. In the entire cohort, corticosteroids have been associated with an increased risk of mortality (OR 2.3, IC 95% 1.3–4.0, *p* = 0.004). In each group, corticosteroids did not show better outcomes. Similarly, they did not show better results among patients without comorbidities or in groups of patients stratified by PRISM. The authors concluded that the stratified risk analysis failed to demonstrate the benefit of any type of corticosteroid in this cohort [10].

Alkhalaf et al., in a retrospective cohort study, assessed that children treated with steroid corticosteroids showed a higher sequential organ failure assessment (SOFA) score and required longer ventilator and inotropic support. However, in-hospital mortality was not significantly different between groups. Patients treated showed a minor length of stay in the PICU. The authors also stated that after adjustment for baseline characteristics, severity scores and medical intervention, no association was found between hydrocortisone and increased mortality (*p* = 0.492) [Table 1] [11].

### 3.2. Shock Severity

The 2019 Cochrane review states that corticosteroids reduce length of stay in PICU for all the participants (average difference—1.07 days, 95% CI from −1.95 to −0.19, 7612 participants, 21 studies, high degree of certainty) and length of hospital stay in general for all participants (average difference—1.63 days, 95% CI from −2.93 to −0.33, 8795 participants, 22 studies; evidence of a high degree of certainty). The dose and duration of therapy did not affect the response to corticosteroids.

In the study conducted by Atkinson et al., previously cited, patients treated with corticosteroids had a higher organ failure degree, a greater severity of disease, and a greater demand for vasoactive drugs than those who did not receive corticosteroids. For the entire cohort, corticosteroids have been associated with a complicated course (OR 1.7, IC 95% 1.1–2.5, *p* = 0.012).

Nichols et al. argue that children with serum cortisol < 18 mcg/dl exhibit a lower severity of the disease than those with >18 mcg/dl serum cortisol (all *p* < 0.05). In children with serum cortisol < 18 mcg/dl, stress dose hydrocortisone therapy was associated with a higher length of stay in the intensive care unit and the hospital and fewer days without ventilatory support (all *p* < 0.05). Children with serum cortisol > 18 mcg/dl treated with hydrocortisone therapy had an increased need for ventilatory and/or inotropic support (all *p* < 0.05).

Alkhalaf et al., in a retrospective cohort study, state that patients receiving corticosteroids had a lower risk of prolonged stay in the intensive care unit than those not receiving them.

### 3.3. Evaluation of Adrenal Function

Weiss and other authors of current guidelines cited several pediatric and adult studies that attempted to use random serum concentrations of cortisol and/or cortisol stimulated with ACTH to identify patients who could benefit more from hydrocortisone therapy, but reliable limit values have not been clearly identified. This is due to various factors, including the variability of the cortisol test itself, cortisol metabolism (11-beta-hydroxysteroid dehydrogenase) during sepsis, corticosteroid binding globulin concentrations, tissue factors (for example, elastase and anti-glucocorticoid compounds) and cellular (for example, glucocorticoid receptor). Therefore, they concluded that the use of random cortisol or stimulation tests to guide the prescription of hydrocortisone in pediatric septic shock cannot be recommended.

Menon et al. instead concluded their study by stating that random levels of free and total cortisol are strongly correlated with the severity of disease in children with septic shock [12].

Vila Perez et al. recommend the administration of hydrocortisone in patients with adrenal insufficiency confirmed by the stimulus test or suspected in cases of shock refractory to inotropic treatment. However, limitations of this test for the diagnosis of corticosteroid insufficiency related to critical diseases and the benefit of corticosteroids both in patients who responded and in those who did not respond suggest that this test should not be used to select patients who will likely benefit from corticosteroids [13].

Singh et al. evaluated adrenal function by dosing basal salivary cortisol and stimulated by ACTH in 51 children with nonresponsive septic shock (30 and 60 min) and basal salivary cortisol in 79 controls. Basal salivary cortisol among patients was higher than in healthy children. Survivors and those with catecholamine refractory shock had higher basal cortisol levels but with a statistically insignificant difference. Absolute adrenal insufficiency (basal salivary cortisol < 1.3 nmol/L) was diagnosed in 15.7%. A relative adrenal insufficiency (increased cortisol above basal value after stimulation < 25 nmol/L) in 68.6% of all patients, 71.9% in nonsurvivors and 71.4% in patients with refractory shock [14].

Nichols et al., at the end of the study results set out in paragraphs 1 and 2, concluded that hydrocortisone in catecholamine-refractory pediatric septic shock is related more to the severity of the disease than the levels of serum cortisol and has been associated with worse outcomes, regardless of the levels.

### 3.4. Early Versus Late Therapy

El Nawawy et al., in a randomized prospective trial, included three groups of patients. The first group received corticosteroids in phase three of treatment, according to the current international guidelines. The second group was managed like the first but underwent stimulus testing with ACTH. The third group received corticosteroids at the beginning of fluid therapy. A fourth group was then created, adding patients who needed corticosteroids in the third phase of therapy from the first and the second group. Serum cortisol and plasmatic ACTH concentrations were assessed in all patients. Data showed a shorter shock reversal time in patients receiving steroids at the beginning of treatment compared to those treated at the third stage of treatment (*p* = 0.046). Mortality resulted in nonstatistical differences between groups [15].

### 3.5. Septic Shock and Immunoparalysis

Bline et al. conducted a study involving 102 children, defining immunoparalysis as a reduced TNFα response (*p* < 0.0001). Thirty-one of the participants received hydrocortisone, and these children were more likely to be older (*p* = 0.04), immunocompromised at baseline (*p* = 0.006), have higher PRISM III scores (*p* = 0.0003), higher vasoactive inotrope scores (*p* = 0.0002), and experience more days of multiple organ failure (MOF) (*p* = 0.002). A total of 33 children exhibited immunoparalysis. Hydrocortisone treatment was associated with a longer duration of MOF in children with immunoparalysis after adjusting for covariates (adjusted relative risk [aRR] 3.7, *p* = 0.0006). No such association was found for children without immunoparalysis (aRR 1.2, *p* = 0.67). Additionally, hydrocortisone was linked to a longer duration of MOF in children with both sepsis and immunoparalysis, but not in those with a more intact immune system. The authors concluded that further well-designed studies utilizing immunophenotyping are needed to optimize hydrocortisone strategies for this patient population [16].

Alder et al. compared all patients with septic shock in their study and found that those who had a complicated course exhibited lower levels of glucocorticoid receptor expression in peripheral blood leukocytes. Further analysis suggested that the combination of low glucocorticoid levels and high cortisol was associated with a higher rate of complicated courses (75%) compared to other combinations [17].

### 3.6. Side Effects

Side effects were extensively explored in the 2019 Cochrane review: The authors state that corticosteroids determined increased risk of muscular weakness (RR 1.21, 95% CI 1.01 to 1.44; 6145 participants; 6 studies; high-certainty evidence), but probably not the risk of superinfections (RR 1.06, 95% CI 0.95 to 1.19; 5356 patients enrolled; 25 studies; evidence of moderate certainty). The risk of hypernatremia (high certainty) and probably hyperglycemia (moderate evidence) also increased. Also, moderate evidence shows that there is probably little or no difference in the risk of gastrointestinal bleeding, stroke, and cardiac events. Low-certainty evidence shows that there is little or no difference in the risk of developing neuropsychiatric disorders. No evidence of increased risk of superinfection was found also in the RCT by El Nawawy et al. [15].

## 4. Discussion

### 4.1. State of the Art

The efficacy of steroids in pediatric septic shock lacks definitive evidence and consensus. The pathophysiological reasoning on sepsis and secondary adrenal insufficiency in critically ill patients and the need to stabilize hemodynamics in the event of failure to respond to inotropes would in themselves be valid reasons for the use of steroid therapy, but current evidence in literature is not convincing for the primary outcomes.

In fact, regarding mortality, although the 2019 Cochrane review highlights a reduction in 28-day and in-hospital mortality, benefits on long-term mortality and in patients with CIRCI are not as clear. On the contrary, several other studies show no difference between treated and untreated patients or even an increase in mortality.

Results regarding the severity of the shock are also equally controversial; although in some studies, steroid therapy seems to reduce the length of stay, the influence on factors more relevant to the severity appears to be reduced, such as the duration of ventilatory and inotropic support and the degree of multiple organ failure.

Further controversies exist regarding the evaluation of adrenal function; in fact, according to the studies analyzed, basal, or stimulated hormonal doses do not allow us to identify with certainty the patients who could benefit most from hydrocortisone therapy due to the various factors that contribute to altering reproducibility; there are also studies such as that of Nichols et al. who find more risks than benefits of steroid therapy regardless of serum cortisol levels.

Another factor that some studies call into question with regard to steroid therapy is immunoparalysis, as children who are more weakened from an immune point of view have a worse response (higher share of MOF) compared to children with more intact immune function without taking into account the role that low serum expression of glucocorticoid receptors has in determining failure of steroid therapy.

Finally, regarding side effects, the Cochrane review leads us to be more vigilant, as they are the only ones supported by a good level of evidence on muscle weakness, hypernatremia, and hyperglycemia.

### 4.2. Knowledge Gap and Further Directions

Recent studies unanimously agree on the necessity of conducting a randomized controlled trial to definitively assess the efficacy of steroid therapy in the management of sepsis. A large-scale trial, the “Stress Hydrocortisone in Pediatric Septic Shock” (SHIPSS study, NCT03401398), is currently underway, aiming to enroll 1032 pediatric patients. This study seeks to clarify the role of hydrocortisone in the treatment of sepsis. The trial protocol involves an initial intravenous hydrocortisone bolus of 2 mg/kg, followed by 6 mg/kg every six hours for a maximum duration of seven days, or until inotropic support is no longer required for at least 12 h. The primary outcomes include 28-day mortality and a reduction of more than 25% in the quality-of-life score. Preliminary results from the study are expected to be released in September 2025.

Future research strategies should focus on developing rapid diagnostic tests with high sensitivity and specificity to identify individuals with FRSS and corticosteroid deficiency, who could benefit from corticosteroid treatment. Furthermore, exploring the genomic phenotype of patients could allow for early prediction of severe disease expression in individuals with septic shock before the development of refractory forms. This would help identify those at higher risk for FRSS progression, potentially guiding the early initiation of corticosteroid therapy in this targeted population. Early treatment initiation in septic shock patients at risk for FRSS may yield better outcomes compared to delayed therapy.

### 4.3. Key Messages

What are the practical considerations for clinical use?

1. Fluid resuscitation, inotropic support and antibiotic therapy remain the standard treatment for managing septic shock in pediatric patients.
2. In cases of refractory septic shock, the use of corticosteroids as a third-line therapy should be tailored for each patient.
3. Adrenal insufficiency may occur in cases of shock refractory to catecholamines; although diagnostic tests for adrenal insufficiency may not be sufficiently accurate, the presence of hypoglycemia and persistent hypotension can guide the clinician toward the use of hydrocortisone.
4. The administration of corticosteroids should be carefully considered in immunocompromised patients.

### 4.4. Strength and Limitations

The main limitation of the review is that we incorporated studies of all qualities, so this affects the robustness of our findings. The narrative nature of this review provides a flexible and broad approach to this topic but does not include an exhaustive search of all possible evidence. Different criteria of selection and analysis could also offer other valid points-of-view.

## 5. Conclusions

There are several key issues surrounding the use of hydrocortisone in pediatric septic shock that continue to spark debate. While current guidelines acknowledge its potential role in managing refractory septic shock, there has yet to be a randomized controlled trial (RCT) that provides conclusive evidence. The existing literature does not offer definitive answers regarding the effectiveness of steroid therapy in pediatric septic shock, as its impact on primary outcomes such as mortality and morbidity remains uncertain and controversial. While it is possible that a subset of patients, particularly those with secondary adrenal insufficiency, may benefit from this treatment, the methods for identifying these patients and determining the most appropriate treatment protocol are still unclear and require further exploration.

## Figures and Tables

**Table 1:** Summary of main results from studies selected.

Title	Type of Article	Population	Corticosteroid Therapy	Main Results and Recommendations
Weiss SL et al. 2020 [4]	Guidelines	Birth–18 yr	IV hydrocortisone (NOS, not otherwise specified)	Against IV hydrocortisone to treat responsive septic shock IV hydrocortisone or no hydrocortisone may be used in refractory septic shock
Menon K at al. 2018 [5]	Expert Opinion	Children (NOS)	Corticosteroids (NOS)	Favors steroids (“…reasonable for clinicians to administer adjunctive corticosteroids to patients with severe septic shock with sepsis endotype B, *Purpura fulminans*, and/or who are not responding to aggressive fluid and vasopressor therapy”
Zimmerman JJ. et al. 2018 [6]	Expert Opinion	Children (NOS)	Corticosteroids (NOS)	Against steroids (“…must conclude that the cumulative published literature does not endorse this intervention”)
Annane et al. 2019 [7]	Cochrane review	Mostly just in adults, two studies in children and adults, six just in children	Corticosteroids (various types and protocols)	There is limited data that prevents drawing definitive conclusions about the effects of corticosteroids in children with sepsis. However, they found no evidence to suggest any difference in the response to corticosteroids between children and adults.
Yang et al. 2020 [8]	Metanalysis		Hydrocortisone, methylprednisolone and dexamethasone. Start dose of hydrocortisone: 2–37.5 mg/kg. Length of therapy: 1–7 days.	Regarding the primary outcome, the fixed effect model showed reduced mortality in patients treated compared to the controls, but applying a random effect model results exhibited a negative result. Dose-response analysis showed also that a low dosage (0.6–5 mg/kg) could reduce mortality, a high dosage (5–37.5 mg/kg) could increase it. The authors suggested against corticosteroids for septic shock in children under current medical knowledge.
Nichols et al. 2017 [9]	Cohort, retrospective	1 m–18 yr	Stress dose hydrocortisone (SDH, initial hydrocortisone dose ≥ 50 mg/m^2^ or ≥1 mg/kg… followed by subsequent hydrocortisone dose ≥ 50 mg/m^2^/day or ≥1 mg/kg/day, … any route for one or more doses	SDH therapy in children with catecholamine-dependent septic shock showed a stronger correlation with illness severity than with rSTC (cortisol) levels and was linked to worse outcomes independent of rSTC levels.
Atkinson et al. 2014 [10]	Cohort, retrospective	Children (NOS)	78% hydrocortisone, 16% methylprednisolone, 6% dexamethasone	Subjects who received corticosteroids experienced a higher burden of organ failure, greater illness severity, higher mortality, and an increased need for vasoactive medications compared to those who did not receive corticosteroids. However, the mortality risk based on the PERSEVERE score did not differ between the two groups. Across the entire cohort, corticosteroid use was linked to an increased risk of mortality (OR 2.3, 95% CI 1.3–4.0, *p* = 0.004) and a more complicated course (OR 1.7, 95% CI 1.1–2.5, *p* = 0.012). In each PERSEVERE-based stratum, corticosteroid administration was not associated with improved outcomes. Likewise, corticosteroid treatment did not show improved outcomes in patients with no comorbidities or in subgroups stratified by PRISM scores.
Alkhalaf HA et al. 2023 [11]	Retrospective Case-Control Study	0–14 y	Hydrocortisone (NOS)	Patients who received corticosteroids had a higher total sequential organ failure assessment (SOFA) score (mean ± SD: 5.5 ± 3 vs. 7.1 ± 3.3, respectively; *p* < 0.01) and required more ventilation support (72% vs. 28%, respectively), as well as greater use of inotropes and vasopressors (74% vs. 34% and 32% vs. 6%, respectively). In-hospital mortality was not significantly different between groups (adjusted HR: 2.66; 95% confidence interval [CI]: 0.66–10.28). However, patients who received corticosteroids had a 42% lower risk of staying in the PICU for more than six days compared to those who did not receive steroids (HR: 0.35; 95% CI: 0.13–0.98).
Menon et al. 2018 [12]	sub-study of a randomized controlled trial (RCT) of hydrocortisone vs. placebo in pediatric septic shock (STRIPES—steroid use in pediatric fluid and vasoactive infusion dependent shock)	0–17 yr	Hydrocortisone (NOS)	Random total and free cortisol levels are reflective of illness severity in children with septic shock. Isolated free cortisol dosage does not provide additional information relative to total in pediatric septic shock.
Vila-Perez et al. 2015 [13]	Narrative review	Children (NOS)	Corticosteroids (NOS)	Children are more likely to develop adrenal insufficiency (AI). Hydrocortisone in patients with AI confirmed by an ACTH test or suspected in refractory forms could be recommended. However, this test has some limitations for the diagnosis of CIRCI, so it should not be used to select patients likely to derive benefit from corticosteroids.
Singh et al. 2013 [14]	Observational study	1–12 yr	/	Basal salivary cortisol among patients was higher than in healthy children. Survivors and those with catecholamine refractory shock had higher basal cortisol levels, but with a statistically insignificant difference.
El-Nawawy A, et al. 2017 [15]	Prospective randomized clinical trial	1 m–4 y	Group (C): 32 patients received i.v. stress dose of hydrocortisone (50 mg/m^2^/24 h) followed by continuous infusion for 5 days from admission and weaning of the drug over 5 days	Early corticosteroid therapy might shorten the shock reversal time, not increasing mortality or superinfections.
Bline et al. 2020 [16]	Secondary analysis of a prospective observational study	<18 y	Hydrocortisone (NOS)	Hydrocortisone in children with immunoparalysis and severe sepsis/septic shock to children determined a longer multi-organ failure.
Alder et al. 2018 [17]	Cohort study	<18 y	/	There was no difference in glucocorticoid receptor (GCR) expression between patients with SIRS, sepsis, and septic shock. When all patients with septic shock were compared, those with a complicated course—defined as experiencing two or more organ failures by day 7 or death by day 28—showed lower GCR expression in all peripheral blood leukocytes. Further analysis revealed that patients with both low GCR expression and high serum cortisol levels had higher rates of a complicated course (75%).
Gupta S et al. 2023 [18]	Review	Children (NOS)	NOS	The RESOLVE study and meta-analysis have failed to demonstrate a reduction of shock duration and mortality in pediatric septic shock treated with steroids. Though adult RCTs have shown a beneficial role of steroids (hydrocortisone plus fludrocortisone) in the reduction of mortality, pediatric RCTs are lacking.
Menon K et al. 2022 [19]	Systematic Review and Meta-Analysis	Children (NOS)	/	Strong associations of markers of organ dysfunction with outcomes among septic children support their inclusion in the data validation phase of the Pediatric Sepsis Definition Taskforce.
Menon K et al. 2022 [20]	Editorial	Children (NOS)	Steroids (NOS)	The authors believe that corticosteroids have the potential to influence morbidity and quality of life of pediatric septic shock patients.
Chong SL et al. 2014 [21]	Review	Children (NOS)	Steroids (NOS)	Steroids in this setting remain controversial.
Wang Y et al. 2019 [22]	Systematic review	Children (NOS)	/	VDD (vitamin D deficiency) is a factor to consider in the occurrence and resolution of septic shock.
Menon K et al. 2013 [23]	Systematic review and metanalysis	Children (NOS)	Steroids (NOS)	No difference in mortality between treated and not treated
Agus MSD 2018 [24]	Expert opinion	Children (NOS)	Steroids (NOS)	Favors steroids in refractory shock
Wheeler DS 2017 [25]	Expert opinion	Children (NOS)	Steroids (NOS)	Against steroids (worse outcomes)
Angurana SK et al. 2017 [26]	Letter	Children (NOS)	Steroids (NOS)	Vitamin D could be an underestimated factor in sepsis
O’Hearn K et al. 2016 [27]	Study protocol	Children (NOS)	At the time of enrollment, a 2-mg/kg intravenous (IV) bolus of hydrocortisone will be administered, followed by 1 mg/kg of hydrocortisone IV every 6 h. Hydrocortisone will then be reduced to 1 mg/kg every 8 h and continued at this frequency until all vasoactive infusions have been discontinued for 12 h.	The STRIPES pilot study will assess the feasibility of a larger trial for clinically important outcomes.
Karagüzel G et al. 2014 [28]	Review	Children (NOS)	Steroids (NOS)	Diagnosis and treatment of AI in pediatric critical illness remain controversial.
Zimmerman JJ et al. 2017 [29]	Review	Children (NOS)	Steroids (NOS)	Steroids have biological plausibility to determine clinical benefits, but large, high-quality pediatric interventional trials are lacking.
Ismail J et al. 2017 [30]	Review	Children (NOS)	Steroids (NOS)	Lack of definitive evidence and consensus for steroids in pediatric septic shock.
Santos L et al. 2013 [31]	Review	Children (NOS)	Steroids (NOS)	Hydrocortisone in refractory shock is suggested in children with suspected or proven adrenal insufficiency.
Sankar J et al. 2017 [32]	Observational Study	<17 yr	/	There is a high prevalence of severe vitamin D deficiency in children with septic shock. It seems to be associated with lower rates of shock reversal.
Prabhudesai S et al. 2015 [33]	Prospective, observational study	30 d–18 yr	/	Larger studies are required to evaluate CGM usefulness in the detection of hypoglycemia and hyperglycemia in pediatric septic shock patients.
Maralihalli MB et al. 2013 [34]	Prospective, observational study	Children (NOS)	/	Arterial bicarbonate at admission to intensive care could predict adrenal insufficiency.
Fitzgerald JC et al. 2016 [35]	Review	<16 yr	Steroids (NOS)	Corticosteroid benefits remain unproven and controversial. Authors cite a retrospective observational study by Wong et al. that compared gene expression in children with septic shock who did and did not receive corticosteroid therapy finding that gene expression related to adaptive immunity was more downregulated in patients who received steroids. This study also affirms that corticosteroids were associated with increased mortality in the higher risk subclass of patients so that technology could help to identify a subset of patients who may not respond favorably to adjunctive corticosteroid therapy.
Menon K et al. 2015 [36]	Editorial	Children (NOS)	Steroids (NOS)	A well-designed trial on the use of corticosteroids in pediatric septic shock is required.
Carmean A et al. 2015 [37]	Survey	Children (NOS)	Steroids (NOS)	Sixty-two percent of recipients rated the importance of steroids in the management of pediatric septic shock as greater than four or five. Ninety percent believed adrenal insufficiency (AI) occurs “sometimes” or “often” in septic shock, and ACTH stimulation testing was frequently used. Eighty-five percent agreed that “some should” receive steroids as it improves outcomes, while 9% agreed that “most should”. Sixty-six percent reported that more than 50% of patients with refractory shock are given steroids. Hydrocortisone was used in 100% of recipients, although dosing and duration varied. The main concerns regarding steroid use were hyperglycemia, superinfections, and critical illness myopathy.
Ponnarmeni S et al. 2015 [38]	Observational	Children (NOS)	Steroids (NOS)	VDD was not associated with greater severity of illness or other clinical outcomes.
Wang Y et al. 2020 [39]	RCT	≤14 yr	/	One dose of vitamin D raised 25OHD levels and decreased the incidence of septic shock in children with vitamin D deficiency (VDD) and sepsis.
Khashana A et al. 2016 [40]	Observational	Term neonates	/	Newborn infants with septic shock and therapy-resistant hypotension had elevated DHEA levels, indicating that 3-beta-hydroxysteroid dehydrogenase activity may be limiting cortisol synthesis.
Menon K et al. 2017 [41]	Study protocol	Children (NOS)	Steroids (NOS)	Deferred consent was acceptable in time-sensitive critical care.
Indyk JA et al. 2013 [42]	Pilot prospective study	Children (NOS)	/	The response to exogenous glucocorticoid therapy mediated by glucocorticoid receptors may be limited. However, the residual receptors in these patients still retain functionality and could be targeted by treatments.
Jardine D 2017 [43]	Expert opinion	Children (NOS)	Steroids (NOS)	Need to characterize endotypes with genome sequencing.
Shibata AR et al. 2015 [44]	Prospective observational cohort	Children (NOS)	/	Patients with shock and greater illness severity exhibit lower glucocorticoid receptor expression in CD4 and CD8 lymphocytes. Additionally, glucocorticoid receptor expression does not show a strong correlation with cortisol levels.
Cimbek EA et al. 2021 [45]	Case report	Children (NOS)	Hydrocortisone (stress dose)	Corticosteroid-induced sinus bradycardia is a side effect that typically resolves once corticosteroid treatment is stopped. Hemodynamic monitoring should be considered during stress-dose corticosteroid therapy.
Habeb AM et al. 2013 [46]	Case Reports	Children (NOS)	Hydrocortisone	Familial glucocorticoid deficiency (FGD) is a heterogeneous condition of isolated glucocorticoid deficiency due to adrenocorticotropic hormone (ACTH) resistance. Patients have adrenal failure with normal electrolytes.
Diller CN et al. 2022 [47]	Survey	Children (NOS)	Hydrocortisone	Nearly all centers reported using hydrocortisone for the treatment of refractory shock. Substantial variation in practice exists in diagnosis, dosing and duration of hydrocortisone.
Kanaris C et al. 2023 [48]	Update	Children (NOS)	Hydrocortisone	In refractory shock
Mestiri Y et al. 2023 [49]	Survey	Children (NOS)	Steroids (NOS)	Corticosteroids, in cases of refractory shock, were always considered by 11.8% of the residents and frequently by 28.5%. Up to 58.33% of the residents would consider steroid treatment in cases of refractory shock regardless of the adrenal status. A few responders (8.33%) would prescribe steroids only if suspected of adrenal insufficiency. No resident asked for an ACTH test before.
Babu S et al. 2023 [50]	Review	Children (NOS)	Steroids (NOS)	The role of hydrocortisone in patients with relative adrenal insufficiency or critical illness-induced corticosteroid insufficiency remains controversial… Presently, the routine use of adjunctive corticosteroids for pediatric septic shock is neither refuted nor supported by high-quality investigations. However, stress-dose hydrocortisone is indicated in patients with septic shock or other sepsis-associated organ dysfunction who have acute or chronic corticosteroid exposure, congenital adrenal hyperplasia or other corticosteroid-related endocrinopathies. Until further data are available, hydrocortisone (50 to 100 mg/kg/d) has been recommended for use in pediatric septic shock, including in those with preexisting heart disease who are not responsive to fluid and initial vasoactive resuscitation.
Atreya MR et al. 2022 [51]	Secondary analysis of a prospective observational study	<14 y	/	PERSEVERE could be a useful tool for prognostic enrichment in future pediatric trials for sepsis
Maddux AB et al. 2022 [52]	Observational Study	1 m–<18 yr	/	Children with septic shock represent a high-risk cohort with high-resource needs after discharge.
Dhochak, N et al. 2022 [53]	Editorial Commentary	Children (NOS)	/	Larger studies are needed to establish clearly the role in MODS scores.
Sahoo T et al. 2020 [54]	Guidelines	/	/	See Weiss et al. 2020
Mendelson J 2018 [55]	Review	Children (NOS)	Steroids (NOS)	In refractory septic shock
Podd BS et al. 2017 [56]	Review	Children (NOS)	Steroids (NOS)	Mentions steroids just in case of associated macrophage activation syndrome.
Argent AC 2017 [57]	Editorial	Children (NOS)	Steroids (NOS)	Against steroids
Menon K et al. 2017 [58]	Pilot trial	Children aged newborn to 17 yr	Patients randomized to the hydrocortisone treatment arm received an initial intravenous (IV) bolus of 2 mg/kg hydrocortisone, followed by 1 mg/kg of hydrocortisone every six hours until the patient met stability criteria … for at least 12 h. … Hydrocortisone dosing was then reduced to 1 mg/kg every 8 h until all vasoactive infusions had been discontinued for at least 12 h	Time on vasopressors, days on mechanical ventilation, PICU, hospital length of stay, and rate of adverse events were not statistically different between the two groups.
Prusakowski MK et al. 2017 [59]	Review	Children (NOS)	Steroids (NOS)	In refractory septic shock
Gelbart B et al. 2017 [60]	Systematic review	Children (NOS)	/	Limited data to support fluid bolus in hospitalized children. Randomized controlled trials are needed to evaluate this therapy in resource-rich settings.
Friedman ML et al. 2014 [61]	Review	Children (NOS)	Hydrocortisone is the steroid of choice … Stress doses of hydrocortisone are commonly in the range of 50 to 100 mg/m^2^, or 2 mg/kg if the patient’s height is not available.	Patients who are on systemic steroids, recent history of systemic steroid use, previously documented adrenal or pituitary dysfunction, or present with *Purpura fulminans* should all be given stress dose steroids. Outside these indications, remains controversial the use of stress dose corticosteroids in septic shock.
Levy-Shraga Y et al. 2013 [62]	Review	Children (NOS)	Steroids (NOS)	Clinical trials examining CIRCI among critically ill pediatric patients are still limited and prevalence, diagnostic criteria, and optimal treatment are still controversial.
Menon K et al. 2013 [63]	Survey	Children (NOS)	Steroids (NOS)	Physicians stated that they were more likely to prescribe steroids for septic shock than for shock following cardiac surgery or trauma. A total of 91.4% would administer steroids to patients who had received 60 cc/kg of fluid and two or more vasoactive medications. Thirty-five percent rarely or never conducted adrenal axis testing. Eighty-seven percent stated that the role of steroids needed to be clarified and that 84.3% would be willing to randomize patients into a trial.
Santschi M et al. 2013 [64]	Survey	Children (NOS)	Steroids (NOS)	Most centers (92%) would administer low-dose steroids if the patient were in refractory shock. No center reported waiting for abnormal cortisol test results or performing an ACTH stimulation test before giving steroids. If the patient develops hyperglycemia, 75% would not initiate insulin, 17% would start insulin without a specific protocol, and 8% would begin insulin following a written protocol.
Galletta F et al. 2022 [65]	Review	Newborns (NOS)	Steroids (NOS)	In pediatrics, recent septic shock guidelines suggest that intravenous hydrocortisone may be used in refractory forms. The role of corticosteroids in the management of septic shock is controversial, but they may increase mortality.
Cucinotta et al. 2022 [66]	Review	Newborns (NOS)	/	Due to the extreme variability of clinical presentation of sepsis in newborns, diagnosis is not always straightforward.
